# Supplementary material for: Self-Organizing Global Gene Expression Regulated through Criticality: Mechanism of the Cell-Fate Change
Source: PLoS One. 2016 Dec 20;11(12):e0167912. doi: 10.1371/journal.pone.0167912 (PMC5173342; doi:10.1371/journal.pone.0167912)
Supplement: S1 Fig — This Fig demonstrates that the timing of the genome-state change does not vary with the choice of an initial state (t0) for A) DMSO-stimulated HL-60 cells (cell population; microarray data) or B) mouse embryonic development (single cell; RNA-Seq data). This result confirms the occurrence of the genome-state change at 24h for DMSO-stimulated HL-60 cells (see more section I), and of reprogramming after the middle 2-cell state for mouse embryonic development (see more section II). C) A pulse-like global perturbation in self-organization occurs at 12-18h (Fig 14: DSMO) in DMSO-stimulated HL-60 cells. The breakdown of criticality occurs at 24h for the initial state at t0 = 12h, before the perturbation, but does not occur for the initial state at t0 = 18h, after the perturbation. This suggests that the global perturbation may relate to the first stage of cell-fate determination (process for autonomous terminal differentiation; see subsection (ii) in Discussion). (DOCX) [file pone.0167912.s001.docx]

**Supplementary Figure S1**:

The timing of the genome-state change occurs at the erasure of an initial-state criticality. This figure demonstrates that the timing of the genome-state change does not vary with the choice of an initial state (*t*_0_) for A) DMSO-stimulated HL-60 cells (cell population; microarray data) or B) mouse embryonic development (single cell; RNA-Seq data). This result confirms the occurrence of the genome-state change at 24h for DMSO-stimulated HL-60 cells (see more **section I**), and of reprogramming after the middle 2-cell state for mouse embryonic development (see more **section II**). C) A pulse-like global perturbation in self-organization occurs at 12-18h (**Figure 14**: DSMO) in DMSO-stimulated HL-60 cells. The breakdown of criticality occurs at 24h for the initial state at *t*_0_ = 12h, before the perturbation, but does not occur for the initial state at *t*_0_ = 18h, after the perturbation. Note: In atRA-stimulated HL-60 cells, a significant pulse-like global perturbation in self-organization occurs at 2-4h. We also observed that the independence of the choice of the initial state for erasure of criticality breaks after the global perturbation (*t*_0_ = 4h) (data not shown). These suggests that the global perturbation may relate to the first stage of cell-fate determination (process for autonomous terminal differentiation; see subsection (ii) in **Discussion).**
