# Supplementary material for: Self-Organizing Global Gene Expression Regulated through Criticality: Mechanism of the Cell-Fate Change
Source: PLoS One. 2016 Dec 20;11(12):e0167912. doi: 10.1371/journal.pone.0167912 (PMC5173342; doi:10.1371/journal.pone.0167912)
Supplement: S2 Fig — Complete sandpile transitional analyses of RNA-Seq data (RPKM): Complete sandpile transitional analyses of RNA-Seq data (RPKM) for A) human and B) mouse embryo development from the zygote stage, and C) T helper 17 cell differentiation from naïve CD4+ T cells. Random human and mouse expression matrixes (A and B: last panels), (i,j) (i: number of time points; j: number of RNAs; right panels in the second row) reflect stochastic overall expression (i.e., zero correlation between any time points); random matrixes are generated by random shuffling of the corresponding original expression matrixes to show linear correlation behavior (3 random samplings are shown in different colors), similar to that of the DMSO random expression matrix (microarray data: Fig 3D). The embryo results suggest that reprogramming of the genome destroys the SOC zygote control in early embryo development. In T helper 17 cell (Th17) differentiation, the development of a sandpile-type critical transition is observed between sequential cell states, where near-linear behaviors exhibit at 3h and 6h (first two panels); this supports the fact that the genome-state change occurs at 3-6h (see Fig 7D). (DOCX) [file pone.0167912.s002.docx]

**Supplementary Figure S2**:

Complete sandpile transitional analyses of RNA-Seq data (RPKM) for A) human and B) mouse embryo development from the zygote stage, and C) T helper 17 cell differentiation from naïve CD4+ T cells. Random human and mouse expression matrixes (A and B: last panels), (*i*,*j*) (*i*: number of time points; *j*: number of RNAs; right panels in the second row) reflect stochastic overall expression (i.e., zero correlation between any time points); random matrixes are generated by random shuffling of the corresponding original expression matrixes to show linear correlation behavior (3 random samplings are shown in different colors), similar to that of the DMSO random expression matrix (microarray data: **Figure 3D**). The embryo results suggest that reprogramming of the genome destroys the SOC zygote control in early embryo development. In T helper 17 cell (Th17) differentiation, the development of a sandpile-type critical transition is observed between sequential cell states, where near-linear behaviors exhibit at 3h and 6h (first two panels); this supports the fact that the genome-state change occurs at 3-6h (see Figure 7D).
