# Supplementary material for: Self-Organizing Global Gene Expression Regulated through Criticality: Mechanism of the Cell-Fate Change
Source: PLoS One. 2016 Dec 20;11(12):e0167912. doi: 10.1371/journal.pone.0167912 (PMC5173342; doi:10.1371/journal.pone.0167912)
Supplement: S1 File — The genuineness of the power-law scaling and the existence of collective behavior of gene expression in power-law scaling: We demonstrate that i) the power-law scaling in the scaling divergent behavior (e.g., Fig 4A) is not a statistical artifact and ii) the dynamics of the collective behavior of gene expression exist through interactions among genes in power-law scaling as follows. (DOCX) [file pone.0167912.s003.docx]

**Supplementary S1 File**

Here, we further demonstrate that i) the power-law scaling in the scaling divergent behavior (e.g., **Fig 4A**) is not a statistical artifact and ii) the dynamics of the collective behavior of gene expression exist through interactions among genes in power-law scaling as follows.

We showed that a scaling region in scaling-divergent behavior (**Fig 4A**) does actually exist in the sub-critical state (bimodal frequency distribution in MCF-7 cells [Tsuchiya M, *et al*., 2015]).

**Fig X1** below (for MCF-7 cells) shows that the bimodality coefficient of random samplings of gene expression converges to that of the sub-critical state, which reveals that, in the power-law scaling (**Fig 4A**: MCF-7 cells), a self-similar frequency distribution exists among random samplings of gene expression. This result provides additional supporting evidence to further confirm the existence of scaling behavior in the sub-critical state.

Next, we demonstrated [Tsuchiya M, *et al.,* 2015] that coherent behavior emerges in the ensemble of stochastic gene expression (coherent-stochastic behavior: CSB). The emergent coherent dynamics follow the dynamics of the center of mass (CM):

(i) The oscillatory coherent dynamics (ABS: **Fig X2**) have been shown to have a good correlation with the dynamics of its center of mass (see Algebraic correlation of CSB in [Tsuchiya M, *et al*., 2015], and

(ii) The coherent dynamics in random samplings shown in **Fig X1** converge to the CM of the sub-critical state (**Fig 10A** in **section IV**). Thus, scaling behavior in the sub-critical state stems from CSB through interactions among genes. In **Fig X2**, the dynamics of probability density profiles of gene expression in the sub-critical state represent the dynamics of CSB.

Therefore, the power-law scaling is not a statistical artifact and the collective behavior of expression as CSB through interactions among genes is present in power-law scaling.

**Fig X1.** **Evidence of scaling behavior in self-similarity of random samplings of gene expression:**

**A)** The bimodality coefficient of random samplings (*n*: number of randomly selected gene expressions) of gene expression converges to that of the sub-critical state (*b* = 0.652 at *t* =30min, as an example). *b* is Sarle's bimodality coefficient for a finite sample, and the results show a bimodal or multimodal distribution when *b*> 5/9 (~0.556). An average bimodality coefficient, <*b*>, over 200 repeats is estimated for each random sampling.

**B)** The frequency distribution of an ensemble of randomly selected gene expression (*n* = 100: Brown, 500: Blue, 1000 mRNAs: Orange) from the sub-critical state has a self-similar bimodal distribution to that of the sub-critical state (Red).

**Fig X2. Dynamics of CSB: collective behavior in the sub-critical state (HRG-stimulated MCF-7 cells).** Pseudo-3-D probability density profiles for the regulatory space show that two CESs form a pair to develop a pendulum-like oscillatory system, i.e., a low-expression state (LES) that swings around a high-expression state (HES) (Fig taken from Fig 7 in [Tsuchiya M, *et al*., 2014]; ON: up-regulation; OFF: down-regulation; EQ: zero change). This pendulum-like oscillatory system acting as a pair of CESs is defined as an autonomous bistable switch (ABS). Furthermore, the oscillatory dynamics of ABS are shown to be well correlated with the dynamics of the center of mass of ABS (see the detailed analysis of ABS dynamics in [Tsuchiya M, *et al.,* 2015]). $\varepsilon\left( t \right)$ represents overall expression at time *t*. Note: Shu and colleagues [Shu G, *et al*., 2003] demonstrated, by means of density analysis of noisy gene-expression profiles, the robustness of gene-expression clustering.

**References:**

1. Shu G, Zeng B, Chen YP, Smith OH (2003) Performance assessment of kernel density clustering for gene expression profile data. Comp Funct Genomics 4: 287–299.
2. Tsuchiya M, Hashimoto M, Takenaka Y, Motoike IN, Yoshikawa K (2014) Global genetic response in a cancer cell: Self-organized coherent expression dynamics. PLOS One 9: e97411.
3. Tsuchiya M, Giuliani A, Hashimoto M, Erenpreisa J, Yoshikawa K (2015) Emergent Self-Organized Criticality in gene expression dynamics: Temporal development of global phase transition revealed in a cancer cell line. PLoS One 11, e0128565.
